# Evidence from a Smoking Management Service in a University Teaching Hospital in Dublin, Ireland monitored by repeat surveys, 1997–2022

**DOI:** 10.1016/j.pmedr.2023.102415

**Published:** 2023-09-13

**Authors:** Ana Mattson, Kirsten Doherty, Ailsa Lyons, Alexander Douglass, Mary Kerley, Sinead Stynes, Patricia Fitzpatrick, Cecily Kelleher

**Affiliations:** a Department of Preventive Medicine & Health Promotion, Saint Vincent’s University Hospital, Elm Park, Dublin, Ireland; b UCD School of Public Health, Physiotherapy and Sports Science, Belfield, Dublin, Ireland

**Keywords:** Smoking cessation, Smoking prevalence, Campus smoking ban, Attitudes, COVID-19 trends

## Abstract

St Vincent’s University Hospital (SVUH) has a comprehensive smoking management programme and since 1997 has conducted periodic surveys of inpatients, outpatients, staff and visitors to establish prevalence of smoking and associated attitudes towards the hospital’s smoke-free campus policy pioneered in 2009. We report trends and describe also the online community stop smoking course (SSC) developed more recently in response to COVID-19. A questionnaire examining attitudes and smoking status was administered by census surveys of inpatients, quota or random sub-sample surveys of staff, and quota surveys with outpatients and visitors in the time period of 1997–2018. Chi square test for trend was used. Smoking rates declined in all groups but significantly so in outpatients (19.5% vs. 10%; p < 0.01), visitors (27.4% vs. 9.5%; p < 0.0001) and staff (30.0% vs. 10.8%; p < 0.0001). Use of E-Cigarettes was low in all cohorts. Rates of smoking were borderline higher in inpatients eligible by income for state-funded General Medical Services (33.2% vs 26.8%, p = 0.099). Support for and awareness of the ban increased over time. Demographic and quit data was compared between participants of in-person or online SSC. The online courses were successful with a maintenance of quit rates (End of Course: 54.7% vs. 55.0%, 1 Month: 50.4% vs. 54.0%, 3 Month: 19.8% vs. 22.5%). While the hospital community’s smoking prevalence has decreased over time and attitudes to the smoking ban have been increasingly positive, the campus is not without difficulties in keeping it smoke-free. We continue to advocate for hospital staff support in enacting this flagship initiative.

## Highlights

• European Network of Smoke-free Hospitals (ENSH) gold level award-winning hospital.
• One of few studies reporting smoking prevalence and attitudes of campus-wide smoking ban in a hospital setting over a twenty-five year time period.
• Documented significant downwards trends in active smoking in staff, outpatients, and visitors since campus-wide smoking ban instituted.
• Novel virtual smoking cessation intervention due to COVID-19 successfully implemented.

## 1 Introduction

St. Vincent’s University Hospital (SVUH) is one of Ireland’s leading academic teaching hospitals. It is located in south Dublin and serves a population of 300,000 in a wide catchment area in County Dublin and Wicklow as well as national and regional specialist services. The hospital currently has approximately 3400 staff and 610 inpatient beds. In January 2009, SVUH became the first hospital campus in Ireland to go completely smoke-free, followed subsequently as a national initiative by the Health Service Executive (HSE), the national health service. SVUH received a gold level International European Network of Smokefree Healthcare (ENSH) services award for this ground-breaking public health measure (Fitzpatrick et al. 2009). The purpose of such a ban is three-fold: it sends a clear message to the general community including hospital patients, staff and visitors, that smoking is a substantial contributing factor to ill-health and premature mortality; it promotes proactive clinical management of current smokers whilst in hospital including assistance in quitting; and it minimises the risk at work to passive smoke exposure, including for health care workers. It is well recognised that national and institutional bans have been effective despite posing challenges in implementation (Frazer et al., 2016, Issue 5., Frazer et al., 2016, Issue 2:).

**Fig. 1:**
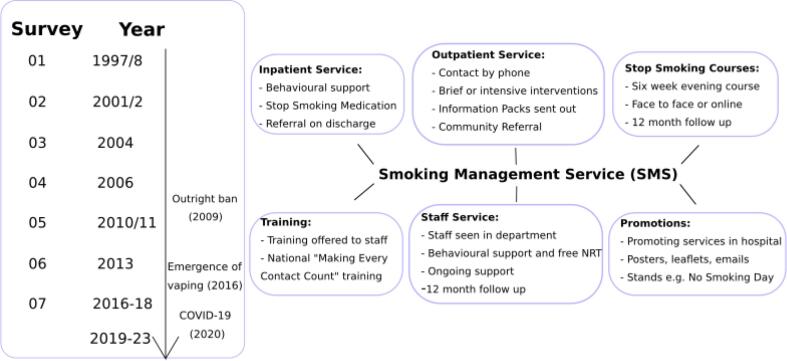
Key components of the SVUH Smoking Management Service (SMS) and timeline of surveys and major milestone events.

Moreover, SVUH had a long track record in health promotion and preventive medicine by that point. The Department of Preventive Medicine and Health Promotion was the first of its kind in Ireland and novel in international terms when first established. A consultant cardiologist had been collaborating with academic colleagues from the associated Faculty of Medicine, University College Dublin on innovative epidemiological studies on cardiovascular disease in a variety of settings including the follow-up study of survivors of unstable angina in the period before by-pass procedures and invasive cardiology became common (Mulcahy et al., 1981). One of the Department’s core functions was a multi-component Smoking Management Service (SMS) which included smoking cessation supports for both in- and out-patients, staff and catchment community (Fig. 1). Since 1997, long before the introduction of the 2009 ban, the Department has been undertaking periodic surveys of inpatients, outpatients, staff and visitors to establish prevalence of smoking and associated attitudes. These surveys serve to monitor trends in the context of both the SMS delivery and external factors that influence use of tobacco. The more recent emergence of E-Cigarettes for instance also poses a challenge (Pougnet et al., 2022, Gallus et al., 2023) and these too are currently banned on our campus and were banned on all hospital campuses by the HSE in 2014. From the outset of the SVUH smoke-free campus policy, it was recognised that for compassionate and logistical reasons, there might be patients who would require an exemption to continue smoking on the hospital campus whilst an inpatient and that a facility should be provided for this purpose, as well as an exemption process agreed by the consultant in charge of that patient’s care. A very significant amount of work was done originally to update the relevant hospital policies for staff and patients, including a vignette training programme for management of scenarios for exemption in hospital and management of difficult cases in implementing the policy (Fitzpatrick et al. 2009). In recent times, exacerbated by the COVID-19 period, significant highly visible smoking has emerged outdoors and there are considerations being given to the reintroduction of monitored smoking shelters again on campus, primarily for health and safety considerations. This would be in keeping with the smoke-free campus exemption policy.

The COVID-19 pandemic also created a significant challenge to supporting ongoing programmes in the context of lockdowns and other infection prevention and control measures that curtailed routine service delivery in-person, including health promotion programmes. As part of the Smoking Mangement Service, Stop Smoking Courses (SSCs) at SVUH have been delivered for over two decades. These courses have traditionally been delivered in-person on the SVUH campus. However, during the COVID-19 pandemic, these courses have embraced telehealth and were delivered online starting in September 2020.

There is evidence that group interventions are more effective than less intensive interventions in helping smokers to quit (Stead et al., 2017). At the SSC smokers are supported to stop smoking and sustain their quit attempt by working through a structured smoking cessation course with the added benefit of group comradery and support. Participants share their experience of quitting in a safe, friendly and confidential environment. Although the course duration is six weeks, participants are followed up until 12 months post-quit. The SSC is open to any smoker wanting support, counselling and information on quitting smoking, and is offered four or five times a year. The Department actively recruits participants to the course from the local community through contacting local employers and voluntary and community groups in the area with information on the courses as well as advertising posters in local train stations, libraries, dentist and general practice offices, shops, and in local newspapers.

To date, we are not aware of any research including twenty-five years of data from a Smoking Management Service in a major hospital setting. In this paper we report trends in the prevalence of smoking and attitudes toward and awareness of the 2009 campus-wide smoking ban in periodic surveys from 1997 to 2018. We also describe the online stop smoking course developed in response to the pandemic and report on the comparison of quit outcomes and patient demographics between those engaged with in-person and online SSCs.

## 2 Materials and methods

### 2.1 Smoking surveys and introduction of campus ban

The methodology for the surveys at SVUH has been described in detail previously (Fitzpatrick et al. 2009). In brief, a short cross-sectional survey was designed for interviewer administration with core questions on smoking status and demographic information; these surveys were administered to different groups within the hospital population: inpatients, outpatients, staff, and visitors. Across the years core questions were included. Smoking status was distinguished between ‘current smoker’, ‘ex-smoker’, and ‘non-smoker’ in all surveys. Other questions included the impact of smoking regulations related to national policies, particularly the outright ban introduced in 2009 and the introduction of E-Cigarettes to this ban. For awareness of the ban, it was asked “Are you aware of this campus-wide smoking ban?” For support for the ban, it was asked “Do you agree with the ban?”. A question on entitlement for the state-funded General Medical Services (GMS) card was asked as a proxy for evaluation of deprivation and socioeconomic status. The survey questions were kept consistent across the years with some minor modifications and additions. In the three most recent surveys (2016–2018), a question assessing the knowledge and use of E-Cigarettes was asked and current use of E-Cigarettes was determined. Coding of branched questions was undertaken. All questions were closed.

Surveys were conducted across seven time periods, grouped by period to reflect the ethical approval process sought and received and for ease of trend analysis: 1997–8, 2001–2, 2004, 2006, 2010–11, 2013 and 2016–18. At all seven time periods, a census survey of inpatients was undertaken by departmental staff in a fixed time window with support primarily from health sciences undergraduate and graduate students. On some occasions this was done across a single day and on others over a fixed number of days but all inpatient surveys were in-person interviews. All inpatients were eligible for interview, provided they were medically fit to participate, but with the right to refuse participation. The number of beds in active service varied over time.

In five of the time periods, staff surveys were also undertaken. These were conducted both in-person and by phone. Either random sub-samples of staff or quota samples controlled to achieve respondents across all employment categories of medical, nursing, allied health, administrative and support staff. On two more recent occasions, in-person quota surveys were also conducted with both outpatients and visitors to the campus, primarily to assess knowledge of and support for the outright campus-wide smoking ban. All surveys were approved by SVUH Research and Ethics Committee.

Chi-square test for trend was used to examine the trends in smoking prevalence in inpatients and staff generally; Pearson’s chi-square test was used to compare proportions in outpatients and visitors over two surveys and to compare smoking prevalance in those entitled to GMS. SPSS version 27 and WinPepi were used for analysis.

### 2.2 Stop smoking courses for SVUH community

The various components of the SMS at SVUH are summarised in detail in Fig. 1. All components of the service had to be reviewed during the period of the pandemic but the community SSC presented particular challenges. The SSCs are targeted at the local community of SVUH and have been running for over two decades, with content reflecting national HSE programmes with 7 weeks of 1.5 h focused sessions on preparing and planning to quit by examining current smoking behaviours, learning about stop smoking medications, stress reduction, and sessions on healthy eating and physical activity. In 2015, there was a slight change in curriculum whereby the course converted to 6 weeks with weekly 1.5 h sessions. Facilitators complete national smoking cessation training and have experience in community, patient, and staff smoking cessation interventions. In September 2020, these courses were converted to an online format through WebEx due to the COVID-19 pandemic. The course content remained consistent to the in-person courses with the exception of carbon monoxide monitoring.

Comparison of in-person and online participant demographics and quit rates was undertaken. Registration and follow-up data were analysed from January 2015 – February 2020 (in-person group 6-week course; 272 participants) and September 2020 – April 2022 (online group 6-week course; 87 participants). Information on demographics such as socioeconomic status, age, sex, use of stop smoking medications and attendance were collected upon registration to the course and analysed upon completion of the course. In addition, follow-up quit data was recorded and analysed at various time points: those who had quit by the end of course, at 1 month, and at 3 months. Evaluation forms were self-administered at the last session of the course. Participants had to have attended at least one session from 2015 onward to be included in analysis. Evaluation data were also available for 127 in-person and 48 online participants. SPSS version 27 and WinPepi were used for analysis.

## 3 Results

### 3.1 Smoking surveys and introduction of campus ban

All inpatient surveys had representative response rates: 98% (711/726; 2 surveys) in 1997/1998, 97% (329/338) in 2001/2002, 94% (259/276) in 2004, 81% (295/365) in 2006, 71% (174/246) in 2010–2011, 67% (182/273) in 2013, and 75% (222/296) in 2016. Eligible participants excluded unoccupied beds and those deemed by nursing staff as too ill to participate. The prevalence of smoking at SVUH from 1997 to 2018 is reported in Table 1. Amongst inpatients the prevalence of smoking fluctuated, from 24.5% in 2004 to 15.1% in 2016 but the generally downwards trend was of borderline statistical significance (p = 0.07). Reported rates amongst staff have been almost uniformly downwards, most recently 9.5% (p < 0.0001). The rates in outpatients halved between the two surveys (p = 0.006) and reduced significantly among visitors also (p < 0.0001).

**Table 1:** Prevalence of Smoking at Saint Vincent’s University Hospital (SVUH) 1997–2018.

Survey Period	Years	Inpatient (%) Total respondents (N)	Staff (%) Total respondents (N)	Outpatient (%) Total respondents (N)	Visitor (%) Total respondents (N)
1	1997–8	24.2	27.4		
		(N = 711)	(N = 365)		
2	2001–2	15.5	17.2		
		(N = 329)	(N = 557)		
3	2004	24.5			
		(N = 257)			
4	2006	22.7	17.8		
		(N = 295)	(N = 225)		
5	2010–11	18.0	10.7	19.5	30.0
		(N = 183)	(N = 300)	(N = 200)	(N = 200)
6	2013	21.4			
		(N = 182)			
7	2016–18	15.1±	9.5*	10.0**	10.8*
		(N = 179)	(N = 263)	(N = 219)	(N = 213)

* p < 0.0001, ** p < 0.01, ± p = 0.07.

After the ban was introduced, there was strong support for the campus smoke-free policy, which varied however according to smoking status with 64.7% of smokers in agreement compared to 92.5% of non-smokers and 89.7% of ex-smokers (p = 0.002). Awareness of the policy was uniformly high, at nine out of ten respondents. Awareness of the outright ban rose significantly amongst inpatients between 2010 (79.2%) and 2016 (91.3%) (p < 0.001). In the most recent surveys in 2016–18, inpatients (91.3%), staff (85.9%) outpatients (92.7%) and visitors (95.3%) all reported high awareness.

In Survey Period 7, a question regarding the awareness and use of E-Cigarettes was included. Among 218 inpatients, just 2% were current users (three patients: two smokers and one ex-smoker), all aged 30–59 years. Inpatients also had a low awareness of E-Cigarettes compared to the staff and outpatient cohorts, with just 47% aware of the devices compared to about 97% in both staff and outpatient groups. Among the 269 staff, more males (11% vs 6%) and more younger staff (10% <50 years vs 6% ≥50) had used E-Cigarettes. Outpatients had the highest prevalence of those currently using E-Cigarettes at 3.7%. Use decreased with increasing age in both inpatients and staff.

In Survey Periods 3–7, inpatient entitlement to the state-funded GMS card was examined. This scheme is a good indicator of relative social deprivation. Over these periods the age of automatic eligibility varied and as a result, entitlement by income was examined only for those under age 65 across periods. We excluded older patients because this group is entitled to a number of free care options based on age but these are not necessarily all means tested. Rates of GMS entitlement in the inpatient population was high at 54.2% average, compared with national rates of approximately 30% (Statista 2022) (Table 2). There was no significant difference in smoking prevalence within individual surveys or overall. However, there are non-significantly higher smoking rates in those entitled to the GMS card (Table 2) than the general inpatient population (Table 1).

**Table 2:** GMS Entitlement and Smoking Prevalence of Inpatient Population in Periodic Surveys.

Survey Period	Inpatients Eligible for GMS Card < 65 y/o (%) Total Respondents (N)	Smoking Prevalence GMS (%) Total Eligible (N)	Smoking Prevalence non-GMS (%) Total Ineligible (N)	p-value
3	69.4	34.0	27.3	0.425
	(N = 144)	(N = 100)	(N = 44)	
4	49.3	41.2	27.1	0.082
	(N = 138)	(N = 68)	(N = 70)	
5	48.8	26.2	34.1	0.425
	(N = 86)	(N = 42)	(N = 44)	
6	44.7	33.3	28.8	0.640
	(N = 94)	(N = 42)	(N = 52)	
7	52.7	26.5	15.9	0.213
	(N = 93)	(N = 49)	(N = 44)	
**Total**	54.2	33.2	26.8	0.099
	(N = 555)	(N = 301)	(N = 254)	

### 3.2 Stop smoking courses for SVUH community

Online participants were significantly more likely to be female, have a mean age over four years younger, and were significantly more likely to use pharmacotherapy (Table 3). There was no significant difference between the online and in-person groups regarding attendance at five or more sessions but attendance of the in-person course was non-significantly better on average (3.8 sessions vs 3.4 sessions; p = 0.087). In the early days of the pandemic, attendance rates were non-significantly higher for the online courses than for the previous in-person courses (attendance at five or more sessions: 58.8% online vs 42.1% in-person; p = 0.06). However with lower session attendance in the latter half of 2021 and early 2022, as restrictions were lifted, the difference between groups has reduced (Table 3).

**Table 3:** Demographic and quit data of in-person and online course participants in Stop Smoking Courses at SVUH 2015–2022.

Variable		In-person (N = 272)	Online (N = 87)	p-value
**Demographic Data**				
Age	Mean (St. Dev)	50.9 (12.7)	46.3 (7.4)	p = 0.002^a^
	Median (Range)	51.0 (23–80)	46.0 (32–71)	
Sex	Female (N (%))	162 (59.1)	63 (72.4)	p = 0.002**^b^**
	Male (N (%))	113 (40.9)	24 (27.6)	
Drug Treatment Status	Drug Tx* (N (%))	120 (43.5)	62 (71.3)	p < 0.001**^b^**
	E-Cigarette Only (N (%))	12 (4.3)	12 (13.8)	
	No Drug Tx* (N (%))	119 (43.1)	12 (13.8)	
Attended 5 or more sessions	Yes (N (%))	114 (42.1)	27 (31.4)	p = 0.078**^b^**
Years Smoking	Mean (St. Dev)	31.6 (12.3)	27.0 (9.1)	p = 0.003**^a^**
	Median (Range)	30.0 (2–65)	27.5 (3–60)	
Heard about the Course over the Internet	Yes (N (%))	39 (14.2)	41 (47.1)	p < 0.001**^b^**
**Quit Data**				
Quit at End of Course	Yes (N (%))	143 (55.0)	47 (54.7)	p = 0.96**^b^**
Quit at 1 Month from Quit Date	Yes (N (%))	76 (54.0)	28 (50.0)	p = 0.78**^b^**
Quit at 3 Months from Quit Date	Yes (N (%))	48 (22.5)	16 (19.8)	p = 0.61**^b^**

*Tx: treatment with Nicotine Replacement Therapy (NRT) or Champix, ^a^ = independent *t*-test, ^b^ = chi-square.

There was no significant difference in quit rates between courses at the end of course, one month, and three month follow-ups. This is a change from initial analysis in the first quarter of 2021 at which point online participants were significantly more likely to remain quit one month from quit date (56.3% vs 35%; p = 0.02). The onsite cohort had a significantly higher average number of years smoking. Valid denominators for variables vary as all questions were optional to participants.

The response rates for evaluation forms were 46.3% and 54.0% for the in-person and online cohorts, respectively. “Useful” ratings were nearly identical between the groups, with 97.6% of the in-person cohort and 97.9% of the online cohort finding the course useful. All online participants felt they had adequate time to talk.

## 4 Discussion

There are very few studies we are aware of that demonstrate continual monitoring of active smoking in the acute hospital setting with the same metholodogy over a twenty-five year period. An added strength of our hospital is that we have a multi-component SMS which can respond formatively to changing drivers of smoking habit in the population. Since our last comprehensive report at the time of the introduction of the campus-wide ban in 2009 we now report both trend data and also the modification of one of our key services in the community in response to the most recent impact of the global pandemic.

This comprehensive report shows that smoking rates have generally been declining over time in the period 1997–2018, in keeping with the general population trends (Healthy Ireland 2018). This is true of all hospital campus users, both inpatients, outpatients, staff and visitors. Staff rates at the last survey are especially low compared to the 2018 general population rate of 20%. The prevalence of smoking in staff was higher than the inpatients in the early surveys, which likely reflects an older catchment area population, whereas the staff are a more mixed demographic and younger. We are not aware of any other Irish hospital having this level of smoking data on hospital inpatients, but we cite a recently published paper that includes inpatient smoking prevalences across Irish hospitals (Fitzpatrick et al., 2023). We also cite two systematic reviews on the impact of smoking bans, the second of which examines the effect of smoking policies in hospitals on smoking rates (Frazer et al., 2016, Issue 5., Frazer et al., 2016, Issue 2:).

These changes in smoking rates are unlikely to be due to smokers not presenting to the campus as a result of the ban. All public hospitals in Ireland shortly followed suit of SVUH’s 2009 ban and it is expected by patients and visitors alike that smoking is prohibited on these premises. The reported support from the SVUH staff and community for, and awareness of, the outright campus-wide smoking ban is also important. This is in keeping with global trends as tobacco continues to pose a major public health challenge.

There was no significant influence of GMS eligibility on inpatient smoking prevalance, however rates were uniformly higher than the general inpatient population for each respective survey period. The prevalence of inpatients who are entitled to a GMS card in our hospital (∼54%) is higher than the general population, which is about 30% (Statistica 2022). This is likely due to the fact there is a private hospital on the same campus as the public hospital, therefore patients who have the ability to pay for private insurance are more likely to seek care at the private hospital. This disparity in smoking status by deprivation is seen nationally and is an area of concern for the national tobacco-free strategy (Healthy Ireland 2019).

Awareness of E-Cigarettes increased over the Survey Period 7 and was highest in staff. E-Cigarette use is low in staff and inpatients, but slightly higher in outpatients/visitors. The higher use in outpatients/visitors is more reflective of the national picture in Ireland with an estimated 4.3% of the population being current users in 2019–2020 (HRB National Drugs Library 2022). Detailed long-term follow-up addressing safety concerns of E-Cigarettes remains ongoing and as a result, these devices are still banned on campus.

The recent COVID-19 pandemic presented major healthcare delivery challenges also, but we report here a successful transition to an online delivery format for our community SSC. The online courses evaluated well and as a result will continue to be offered in the future although in-person courses will also be offered, to ensure we are not excluding those for whom the online format is unsuitable. There are broader lessons for the future also, as people have become more familiar with online consultation and care and home testing, including rapid antigen diagnostic tests. The move online maintained quit rates and produced similar “useful” ratings. Evaluation forms were completed in the final week so there is a potential for results to be skewed by those who had been successful at quitting in the course, despite efforts to prompt those who had dropped out of the course to complete it as well. Technology requirements may have deterred older smokers. The awareness of the link between smoking and COVID-19 severity may have initially positively impacted quit rates. Lack of carbon monoxide monitoring did not appear to alter effectiveness of these online groups. Fatigue with virtual environments due to the ongoing pandemic and society opening back up could have resulted in lower attendance more recently online.

Our experience with a smoke-free campus is not unique. Fu et al. (2022) report that compliance with non-smoking outdoors on a hospital campus can be hard to achieve. Garritsen and colleagues in association with smoke-free policies also saw a decline in smoking prevalence (Garritsen et al. 2022). Across the United States of America (Goldstein et al., 2009, Williams et al., 2009) hospital campus bans were widely introduced around the same time as ours in Ireland. This goal is achievable globally. Hoffman and Tan (2015) conducted an overview of systematic reviews on the health-related effects of government tobacco control policies and concluded that smoking bans and price increases of products were effective tobacco control measures.

## 5 Conclusions

This report presents novel findings from twenty-five years of monitoring smoking status of our hospital population, implementing and maintaining a groundbreaking campus-wide smoking ban and recently, adjusting our practice to the online setting due to the COVID-19 pandemic. This is a flagship initiative for the catchment community in keeping with the World Health Organisation Health Promoting Hospital concept (International Network of Health Promotion Hospitals and Health Services 2022).

We report strong trends in positive attitudes towards this ban, a reduced smoking prevalence in all cohorts, a tendency towards higher rates of smoking in inpatients of lower socioeconomic status, and an effective virtual stop smoking course for our community catchment area. We will continue to monitor trends with another round of surveys when infection prevention and control considerations permit. We continue to strongly advocate for the support of clinical staff and human resources to retain the exemption system for patients, reinforce to staff that smoking on campus at work remains prohibited, refer to the Smoking Management Service run through our department and reinforce the messaging to all coming on campus that this remains a pioneering smoke-free hospital.

## Funding

This research received no external funding.

## Institutional Review Board Statement

The study was conducted according to the guidelines of the Declaration of Helsinki, and approved by the Ethics Committee of St. Vincent’s University Hospital. Ethics No: RS16-002.

## Informed Consent Statement

Informed consent was obtained from all subjects involved in the prevalence studies**.**

## Data Availability Statement

All data collected is held locally within SVUH servers. Data is not available outside of the hospital.

## CRediT authorship contribution statement

**Ana Mattson:** Conceptualization, Methodology, Formal analysis, Writing – review & editing, Project administration. **Kirsten Doherty:** Conceptualization, Methodology, Formal analysis, Writing – review & editing, Project administration. **Ailsa Lyons:** Conceptualization, Methodology, Formal analysis, Writing – review & editing, Project administration. **Alexander Douglass:** Conceptualization, Data curation, Writing – review & editing. **Mary Kerley:** Conceptualization, Writing – review & editing, Project administration. **Sinead Stynes:** Conceptualization, Writing – review & editing, Project administration. **Patricia Fitzpatrick:** Conceptualization, Methodology, Formal analysis, Writing – review & editing. **Cecily Kelleher:** Conceptualization, Methodology, Formal analysis, Writing – original draft, Writing – review & editing.

## Declaration of Competing Interest

The authors declare that they have no known competing financial interests or personal relationships that could have appeared to influence the work reported in this paper.

## Data Availability

The data that has been used is confidential.
